# Flourish of Proton and Carbon Ion Radiotherapy in China

**DOI:** 10.3389/fonc.2022.819905

**Published:** 2022-02-14

**Authors:** Yue Li, Xiaoman Li, Jiancheng Yang, Sicheng Wang, Meitang Tang, Jiawen Xia, Yunzhe Gao

**Affiliations:** ^1^ Institute of Modern Physics, Chinese Academy of Sciences, Lanzhou, China; ^2^ Department of Radiation Medicine, School of Basic Medical Sciences, Peking University Health Science Center, Beijing, China; ^3^ Huizhou Research Center of Ion Science, Chinese Academy of Sciences, Huizhou, China; ^4^ School of Nuclear Science and Technology, University of Chinese Academy of Sciences, Beijing, China

**Keywords:** radiotherapy, proton, carbon ion, clinical trial, radiation oncology, proton, tumor

## Abstract

Proton and heavy ion therapy offer superior relative biological effectiveness (RBE) in the treatment of deep-seated tumors compared with conventional photon radiotherapy due to its Bragg-peak feature of energy deposition in organs. Many proton and carbon ion therapy centers are active all over the world. At present, five particle radiotherapy institutes have been built and are receiving patient in China, mainly including Wanjie Proton Therapy Center (WPTC), Shanghai Proton Heavy Ion Center (SPHIC), Heavy Ion Cancer Treatment Center (HIMM), Chang Gung Memorial Hospital (CGMH), and Ruijin Hospital affiliated with Jiao Tong University. Many cancer patients have benefited from ion therapy, showing unique advantages over surgery and chemotherapy. By the end of 2020, nearly 8,000 patients had been treated with proton, carbon ion or carbon ion combined with proton therapy. So far, there is no systemic review for proton and carbon ion therapy facility and clinical outcome in China. We reviewed the development of proton and heavy ion therapy, as well as providing the representative clinical data and future directions for particle therapy in China. It has important guiding significance for the design and construction of new particle therapy center and patients’ choice of treatment equipment.

## Introduction

According to the latest Statistics on Cancer, there were an estimated 19.29 million new cases and 9.96 million cancer deaths worldwide in 2020 (1). Among them, 4.57 million new cancer cases and 3 million deaths were reported in China. Surgery, radiotherapy (RT) and chemotherapy are the standard methods of cancer treatment (2). RT is one of the most effective manners for the treatment of primary and metastatic solid tumors, microscopic tumor extensions, as well as regional lymph nodes. Conventional radiotherapy (photon or electron) has some disadvantages because of the limitation of tumor location, beam arrangement, technique and modality (3, 4). It is difficult to avoid radiation exposure to important organs and tissues around the tumor, resulting in certain short-term and long-term complications and sequelae. These radiation complications seriously affect the quality of life of patients. In order to ensure that the radiation dose of normal organs and tissues around the tumor does not exceed the tolerance dose, the radiation dose to target has to be reduced, resulting in the reduction of local control rate (5).

In recent decades, particle radiotherapy has been developed in clinical practice in United States, Japan, Germany and other countries, including proton beam and heavy ion (mainly using carbon ions). According to data released by Particle Therapy Co-Operative Group (PTCOG), more than 290,000 patients have been treated worldwide with particle therapy by the end of 2020, including almost 250,000 with protons and 40,000 with carbon-ions (6). Consensus Guidelines for pencil-beam scanning proton beam therapy (PBT), particularly intensity modulated PBT for thoracic malignancies and for Prostate Cancer mark the growing acceptance of proton therapy as a standard treatment for these tumor (7, 8). Fascinated by those results, China began to explore the development of proton radiotherapy since 1996. In the past more than 20 years, several proton projects have been put on the agenda, but some of them have been stopped due to capital chain rupture or high maintenance cost. Even so, several particle radiotherapy institutes have been built and are receiving patient in China, mainly including WPTC (Wanjie, Zi-Bo), Shanghai Proton Heavy Ion Center (SPHIC, Shanghai), Heavy Ion Cancer Treatment Center (HIMM, Wuwei, Gansu), and Chang Gung Memorial Hospital (CGMH, Linkou and Kaohsiung). Ruijin Hospital also established a proton center at the end of 2021. China was the fourth country, of the now five countries, using carbon ion therapy. Recently, ion therapy guideline (Version 2020) based on the latest research data has been formulated, which guides the clinical practice of ion therapy in China and promote the popularization and application of ion therapy (9). However, there is currently no systematic review of particle therapy equipment and treatment outcomes in China. In this review, we will briefly summarize the history and current situation of the development of PBT and carbon ion radiotherapy (CIRT) in China, analyze the clinical cases, and discuss the future development and challenges of proton and heavy ion equipment in China based on national circumstances and the current situation of ion radiotherapy.

## Physical and Radiobiological Basis of Proton ad Carbon Ion Radiotherapy

### Physical Characteristics

The advantages of the proton and heavy ion medical accelerator are mainly reflected in the Bragg Peak distribution of the linear energy transfer (LET) of high-energy particle beams in human tissues. Thanks to this characteristic in human tissues, we can use this property to selectively deposit energy mainly in the tumor sites, while less depositing in normal tissues, so as to eliminate cancer without damaging or less damaging normal tissues (10). Several treatment planning studies between photon, proton, and carbon ion radiotherapy were carried out in China. Those three modalities achieved similar levels of target conformation. However, PBT and CIRT significantly reduced the OARs dose in the treatment of locally recurrent nasopharyngeal carcinoma (LR-NPC) (11, 12).

Although the physical properties of carbon ions and protons are very similar, carbon ion radiotherapy has its own characteristics compared with protons. (i) transverse scattering and range straggling are relatively small, which means smaller beam halo (13); (ii) Compared with proton beams, heavy ion beams require higher energy to reach deep tumors. Therefore, manufacturing carbon ion equipment requires larger accelerators and beam delivery systems (14).

### Radiobiological Characteristics

**Figure 1:**
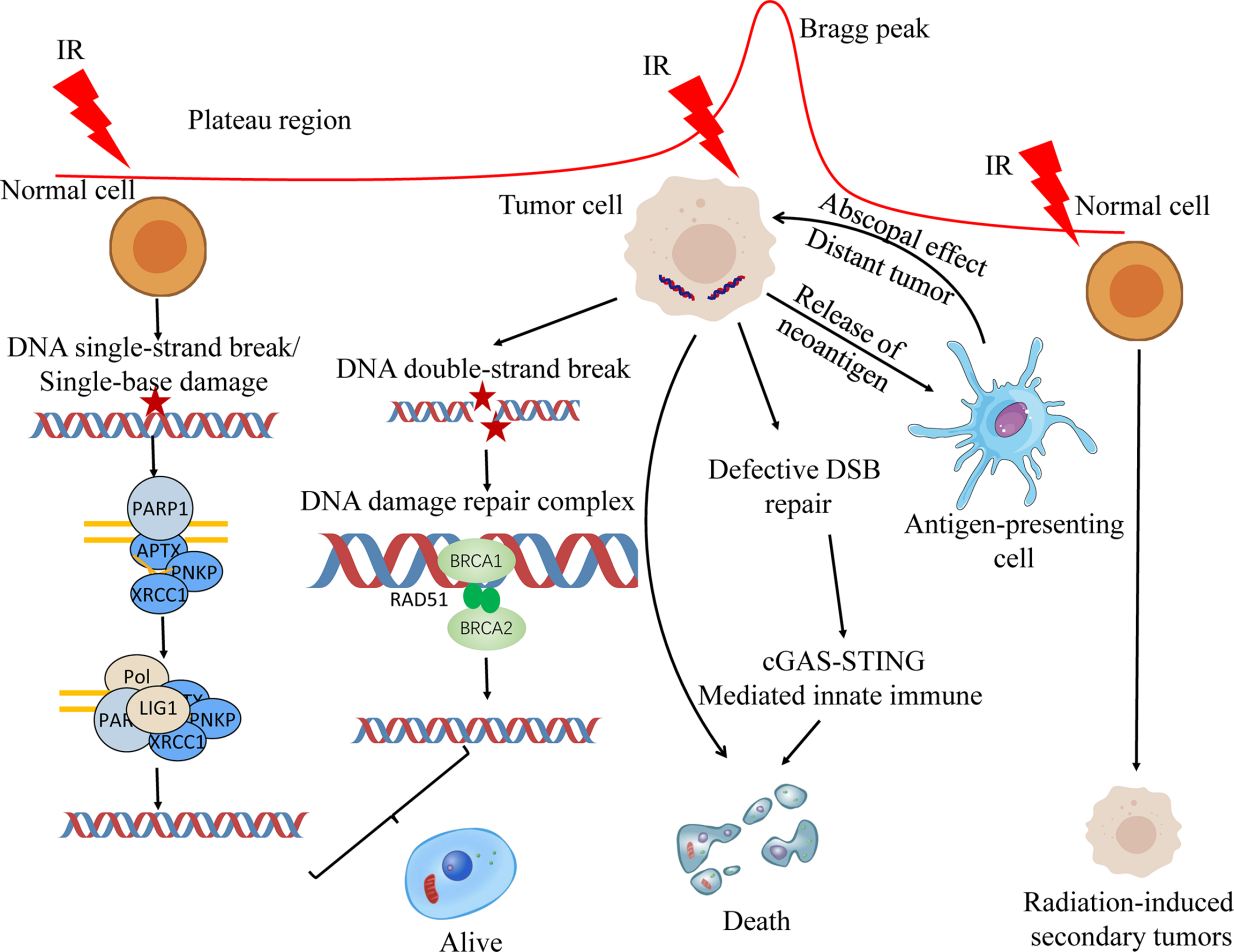
Biological principles of proton and carbon ion radiotherapy. Ionizing radiation primarily causes single-base damage, single strand break damage, and double strand break damage. Proton and carbon ion beams can cause serious damage to tumor cells by their unique Bragg peak, and most of the cells can’t be repaired in time, resulting in apoptosis. The first two types of damage are mainly repaired by the base excision repair (BER) pathway. homologous recombination (HR) and non-homologous end joining (NHEJ) are both involved in the repair of DNA double-strand breaks (DSBs). On the one hand, radiotherapy can kill the DSB repair-deficient cells; on the other hand, the neoantigens produced by radiotherapy can be processed by lymphocytes to produce lethal effects. At the same time, radiotherapy can promote the release of nuclear and mitochondrial DNA, and cells can increase the play of innate immune function through cGAS-STING mediated innate immune, and jointly promote the death of tumor cells.

Relative biological effectiveness (RBE) is the ratio of absorbed doses of two radiations required to produce the same biologic effect (15). RBE is affected by the ion species, dose, LET, cell and tissue type, biological endpoint and other factors (16). Within a certain range, RBE increases with the increase of LET (17). Compared with photon or PBT, CIRT has a higher LET, and the estimated RBE of CIRT has a 2 to 5 larger times RBE than photon RT depending on the irradiated tissues and cells (18, 19). The Bragg peak region of the carbon ion beam appears at the same position as the RBE peak region, which allows the carbon ion beam energy to act on the target region to the maximum, while the normal tissues that the beam passes through are less affected (20, 21). Carbon ions use their biological properties to cause severe clustered DNA double-strand breaks to kill tumor cells (22) (**Figure 1**). At the front or tail of the Bragg peak, most normal cells are repaired through single-strand or double-strand break repair mechanisms. The detailed mechanism of ionizing radiation induced DNA damage repair has been discussed in previous publication (23). In addition, there are some reports that these radiations, especially secondary neutron, may induce secondary tumors (24).

In addition to high RBE, carbon ions also have a lower Oxygen Enhancement Ratio (OER). OER refers to the ratio of the dose required for hypoxic cells and normoxic cells to produce the same biological effect. Due to the rapid growth of tumor cells, the surrounding necrotic tissue hinders the supply of oxygen, so the tumor cells are in a state of hypoxia. Photons, X-rays, and protons have higher OERs and lower lethality to hypoxic tumor cells than carbon ions (25). At present, there have many pre-clinical radiobiology facilities to do *in-vitro* and *in-vivo* studies in China (**Table 1**).

**Table 1:** List of pre-clinical radiobiology facilities in China.

Institute or University	Location	Type of Beam
Key Lab. Ion Beam Bioengineering (LIBB)	Hefei, China	Proton
NHC Key Lab. of radiobiology	Jilin, China	X-rays, Proton, Carbon ion
Institute of Radiation Medicine	Shanghai, China	Proton, Carbon ion
Institute of Modern Physics	Lanzhou, China	X-rays, Carbon ion
State Key Lab. of Radiation Medicine and Protection	Suzhou, China	X-rays, Proton, Carbon ion
Institute of Heavy Ion Physics	Beijing, China	Proton

## Proton and Carbon Ion Therapy Facilities

Up to December 2021, there are 107 proton and heavy ion therapy centers in operation all over the worldwide based on PTCOG data (26). However, there are only twelve operational carbon ion therapy centers worldwide. The availability of heavy ion treatment is quite limited worldwide due to the high requirement of related technological research and the costly construction of medically dedicated accelerator and support systems (27). The heavy ion equipment is large in size, high in installation and operating costs, and the rotating gantry treatment technology is easier to implement in the proton equipment. At present, the Heidelberg Ion Treatment Center (HIT) in Germany and QST in Japan are the only carbon ion treatment centers in the world with rotating gantry treatment technology (28, 29).

Although there are many proton and heavy ion centers in preparation, only five proton and heavy ion treatment centers are in operation in China (**Table 2**). The good news is that, eleven and ten proton and heavy ion sites are under construction (**Table S1**) or in planning stage (**Table S2**) based on PTCOG data, respectively. In addition to the different particles used in the five particle therapy centers, the main differences are the beam delivery system, rotating gantry, and treatment planning system. Among them, only HIMM is a self-developed medical device in China. In addition, the first proton therapy device (Shanghai Advanced Proton Therapy Device, APTRON) has been independently developed by Shanghai Ruijin Hospital, China (30). Two patients (one for recurrent pituitary adenoma and another for recurrent cranial base chordoma) were treated at Ruijin hospital up to now. As of the date of writing this manuscript, about 8,000 patients in China have received particle therapy.

**Table 2:** Comparison of particle therapy facilities in operation in China.

Items	WPTC (Wanjie Proton Therapy Center)	SPHIC (Shanghai Proton and Heavy Ion Center)		CGMH (Chang Gung Memorial Hospital)	HIMM (heavy-ion medical machine)	Ruijin Hospital
Particle	Proton	Proton	Carbon	Proton	Carbon	Proton
Accelerator Type, Energy range (MeV/*u*)	C 70-230	S 50-250	S 85-430	C 75-235	S 120-400	S 70-235
Maximum depth of incidence (in water)	30 cm	30 cm		30 cm	27 cm	30 cm
Beam directions	1 360°gantry, 1 horiz. fixed beam	3 horiz. fixed beams, 1 oblique beam		4 360° gantries (for PBT)	4 horiz, 1 vertical, 1 oblique	1 ocular beam line, 1 fixed beam, 1 360°gantry, 1 180° half-gantry
Beam delivery system	PBS/scatter scanning	PBS/Raster scanning		PBS/wobbling	PBS/uniform scanning	PBS/scatter scanning
Treatment couch	Robotic Couch	Self-developed 360° rotating treatment chair		6-dimensional Robotic Couch	Robotic Couch	Robotic Couch
Dose monitoring system	Yes	Yes		Yes	Yes	Yes
In-room image-guidance	2D kV X-ray image	2D kV X-ray image		2D/3D CBCT	2D kV X-ray image	2D kV X-ray image
Respiratory Gating System	Yes	Yes		Yes	Yes	Yes
Treatment plan system	Varian Eclipse (Eclipse physical dose (EPD [RBE 1.1])	Siemens Syngo (LEM I)		Varian Eclipse/RayStation (LEM/MKM/LNDM)	self-developed ciPlan (LQ/LNDM)	RayStation 10 (LEM/MKM/LNDM)
Sources of equipment	IBA ProteusPlus	Siemens IONTRIS (Germany)		Sumitomo Heavy Industries (SHI)	Self-developed	Self-developed
Start of construction	2001	2009		2011/2014	2012	2014
Start of treatment	2004	2014		2015/2018	2019	2021
Patients	1829	3259		3109/638	361	2

S, Synchrotron; C, Cyclotron; LEM, local effect model; MKM, microdosimetric kinetic model; LQ, linear quadratic; LNDM, logistic nanodosimetry model. Data collected by the PTCOG (update Jan 2022).

**Figure 2:**
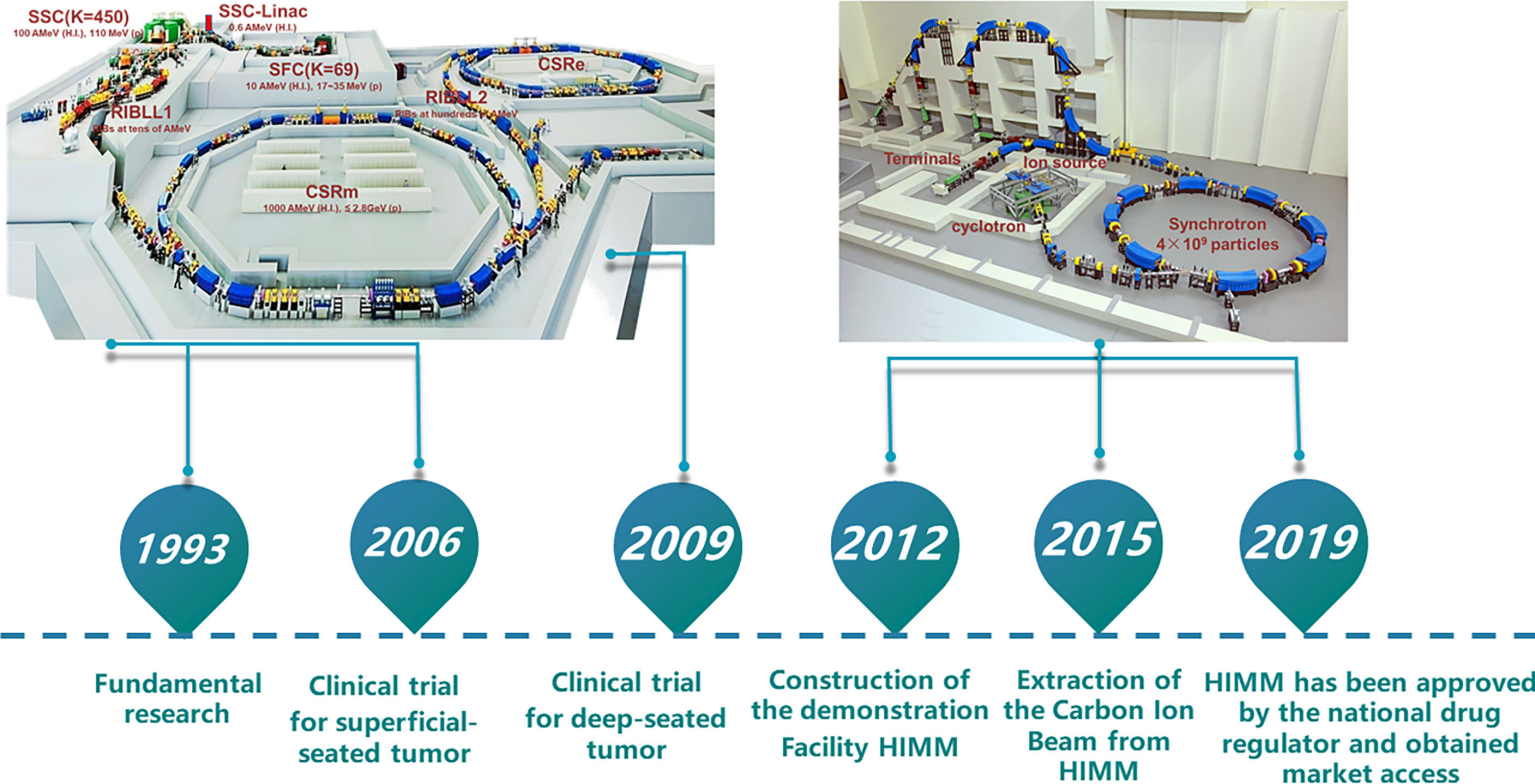
Research roadmap of heavy ion cancer treatment at IMP. The Institute of Modern Physics (IMP) built two cancer treatment terminals on Heavy Ion Research Facility in Lanzhou (HIRFL). Based on continuous research and development of advanced accelerator technology and nuclear detection technology, biological basic research and preliminary clinical trial of CIRT, the IMP has successfully developed HIMM. CSR, Cooler-Storage-Ring; CSRm, CSR main ring; CSRe, CSR experimental ring; RIB, Radioactive Ion Beams; SSC, Separated Sector Cyclotron; SFC, Sector-Focusing Cyclotron; RIBLL1, Radioactive Ion Beam Line 1; RIBLL2, Radioactive Ion Beam Line 2; H.I., Heavy Ions.

Proton and heavy ion therapy have gradually developed in the past two decades in China. In 1989, the Institute of Modern Physics (IMP) established the Heavy Ion Research Facility in Lanzhou (HIRFL), and began basic research work on heavy ion therapy for tumors in 1995. To realize the transformation from bench to bedside, a total of 213 patients were treated with CIRT at the HIRFL of IMP from 2006 to 2013, satisfying efficacy and acceptable toxicities has been obtained in several types of tumors (31). Encouraged by the results from the HIRFL of IMP, a heavy ion therapy demonstration device assigned for clinical use, i.e., the heavy‐ion medical machine (HIMM), has been designed and built independently by the IMP in Wuwei and Lanzhou, Gansu Province, China. In 2019, HIMM completed clinical trials of 47 patients. Since then, proton and heavy ion radiotherapy in China has begun to develop rapidly (**Figure 2**). The WPTC is the first hospital to carry out proton therapy in China in 2004. Due to the expensive maintenance costs of the equipment, the center ceased operations for a few years and reopened in 2015. Shanghai Proton Heavy Ion center (SPHIC) installed the IONTRIS device (IONTRIS device) manufactured by Siemens in Germany. At the request of the China Food and Drug Administration (CFDA), SPHIC conducted a clinical registration trial for IONTRIS to further verify the safety and efficacy of IONTRIS in the treatment of cancer patients. CGMH has built two particle therapy centers in Taiwan, and received patient in 2015 and 2018, respectively. Those significant peer-reviewed publications in clinical, physics, and biology of particle radiotherapy from these centers have been listed in **Table 3**.

**Table 3:** Publication lists in clinical, physics, and biology of particle radiotherapy from these centers (update Jan 2022).

	WPTC	SPHIC	CGMH	HIMM	Ruijin Hospital
Clinical	(32–34)	(18, 35–52)	(53, 54)	(55, 56)	No publications
Physics	No publications	(57–60)	(61, 62)	(31, 63–71)	(72–77)
Biology	No publications	(78–80)	(81)	(82–92)	No publications

Treatment planning system (TPS) is the nerve center or command center in radiotherapy process. TPS refers to software designed to help doctors or physicists design and optimize treatment planning. Currently commercially available treatment planning systems are developed by business companies, such as RayStation (RaySearch Medical Laboratories, Stockholm, Sweden), XiO (Elekta., Stockholm, Sweden), Eclipse (Varian Medical Systems, Palo Alto, CA, USA), and Pinnacle (Philips Healthcare, Andover, MA, USA) (93). Conventional radiotherapy started relatively early in China, and it has gradually matured (94). Proton radiotherapy equipment and radiotherapy planning system started late in China. At present, most of the proton therapy centers in operation in China use the commercial TPS. The Syngo treatment planning software system (versions VC11 and VC13; Siemens Healthcare, Erlangen, Germany) was adopted at the SPHIC, which considers local values of the RBE based on the local effect model (LEM) (18). A self-developed heavy ion radiotherapy treatment planning system was adopted at the HIMM (66, 95).

## Initial Clinical Outcomes of PBT and CIRT in China

Several clinical practices have confirmed potential advantages of PBT and CIRT (96). Although there are only preliminary clinical data, China has also gradually gained experience on the basis of clinical trials. Up to date, fifteen observational or interventional clinical trials are registered in Chinese Clinical Trial Registry (ChiCTR, https://www.chictr.org.cn/) and eighteen clinical trials are registered in ClinicalTrials.gov (https://clinicaltrials.gov/) at SPHIC (**Table 4**). At CGMH, three clinical trials were registered in ClinicalTrials.gov. In HIMM, six clinical trials are recruiting patients, including pancreatic cancer, lung cancer, three for esophageal cancer and muscular-infiltrating bladder cancer in ChiCTR in 2021. It can be seen that large parts of the clinical trials are recruiting patients, and several trials were retracted because of shortage of patients. One possible reason is that the acceptance of particle beam therapy is not enough among cancer patients in China. To assure the reliability, we mainly summarize the clinical results that have been published or reported in this section.

**Table 4:** Clinical trials registered in ClinicalTrials.gov for radiotherapy in China (up to January 2022, data collected from https://clinicaltrials.gov/).

Tumor site	Identifier	Enrollment	Status	Phase
Nasopharyngeal carcinoma (NPC)	NCT04528394	136 (Estimated)	Recruiting	II
	NCT02569788	9 (Actual)	Terminated (Slow accrual of patients.)	I/II
	NCT02795195	55 (Actual)	Active, not recruiting	I/II
	NCT02801487	6 (Actual)	Terminated (Slow accrual of patients.)	I/II
	NCT04533620	96 (Estimated)	Not yet recruiting	II
	NCT04143984	146 (Estimated)	Not yet recruiting	II
Hepatocellular carcinoma (HCC)	NCT02802124	48 (Estimated)	Recruiting	I
	NCT02946138	0 (Actual)	Withdrawn (enrollment was too slow)	II
	NCT02640924	166 (Estimated)	Recruiting	III
Prostate Cancer	NCT02935023	47 (Estimated)	Recruiting	II
	NCT02739659	61 (Estimated)	Recruiting	I/II
	NCT04724577	30 (Estimated)	Recruiting	I
	NCT05010343	140 (Estimated)	Recruiting	II
Pancreatic Cancer	NCT03949933	10 (Actual)	Completed	I
	NCT04082455	49 (Estimated)	Recruiting	II
	NCT03403049	14 (Actual)	Completed	I
Glioblastoma	NCT04536649	369 (Estimated)	Not yet recruiting	III
	NCT02608762	72 (Estimated)	Recruiting	II
H&N ACC	NCT02942693	50 (Estimated)	Recruiting	II
SNMM	NCT05009446	28 (Estimated)	Recruiting	Early I
Lymphoma	NCT03969693	50 (Estimated)	Recruiting	Early I

To register as a medical institution, safety and efficacy have been verified at SPHIC and HIMM. From June 2014 to December 2014, SPHIC completed a phase II clinical trial (IONTRIAL) designed to verify the safety and efficacy of Siemens IONTRIS ion therapy system (97). All 35 patients enrolled in the IONTRIAL trial, and there was no case of grade 2 or above adverse reactions during the treatment and follow-up. No patient death or treatment-related moderate or severe adverse events have occurred in all patients during follow-up (98).

From 2006 to 2008, 103 cases of shallow-seated tumor patients, including skin squamous cell carcinoma and malignant lymphoma, treated at IMP (31). In the next five years, 110 deep-seated tumor patients have been treated with high-energy carbon ions at IMP. The tumor volume was reduced to varying degrees. There was no obvious side-effect in the irradiated site and the whole body after treatment (99).

In order to build a more compact medical accelerator, HIMM was built based on the clinical experience gained in the previous stage of IMP. The clinical trial of HIMM began on November 6, 2018, and was completed on February 25, 2019 in all 46 subjects. At the end of treatment, all 46 patients were evaluated according to the Response Evaluation Criteria in Solid Tumors RECIST Version 1.1. Ninety days after the end of treatment, the local control rate was 100%, and the objective response rate was 28.26%. No adverse events occurred according to the Common Terminology Criteria for Adverse Events (CTC.AE) version 4.03 (100).

## Clinical Practices

Major cancer types treated with particle therapy in China have been summarized in **Table 5**, including treatment modality, number of patients, gender of patients, age of patients, overall survival, local control, late toxicity and treatment center. The detailed description for different cancers has been discussed.

**Table 5:** Major cancer types treated with particle therapy in China.

Tumor sites	Time period	Reference	Treatment	Gender (No. of patients)	Mean Age	Overall survival	Local control	Late ≥GIII	Centers
Prostate cancer	7/2015 – 1/2018	(45)	59.2–60.8 GyE/16 fractions (n = 46)	Male (64)	70.5	Unknown	Unknown	0	SPHIC
			66 GyE/24 fractions (n = 18)						
Chordoma	11/2004 – 11/2008	(32)	50.4-80 GyE/28-40 fractions	Male (18)	41.6	90.3% (3 years)	Unknown	Unknown	WPTC
				Female (13)					
						87.1% (5 years)			
Skull Base Sarcomas	7/2014 – 5/2019	(46)	64–70Gy (RBE)/32–35 fractions	Male (37)	38	91.2% (1 years)	89.2% (1 years)	2	SPHIC
				Female (25)					
						80.2% (2 years)	80.2% (2 years)		
Orbital malignancies	7/2014 - 5/2018	(47)	PBT (56 GyE/28 fractions) followed by CIRT boost (15 GyE/3 fractions)	Male (14)	46.5	100% (2 years)	92.9% (2 years)	0	SPHIC
				Female (8)					
Locoregionally recurrent head and neck malignancies	5/2015 – 11/2017	(18)	60 GyE (range 50–69 GyE, 2.0–3.5 GyE/daily fraction)	Male (101)	49	95.9% (1 years)	84.9% (1 years)	10	SPHIC
				Female (40)					
Sinonasal malignancies	5/2015 - 6/2019	(48)	PBT:60-66 Gy (RBE) in 30-35 fractions	Male (64)	49	82% (2 years)	83% (2 years)	4	SPHIC
				Female (47)					
			CIRT: 63-73.5 Gy (RBE) in 18-21 fractions						
Nasopharyngeal carcinoma	2016–2018	(49)	IMPT:69.96 GyE/33 fractions	Male (64)	47.6	100% (2 years)	94.4% (2 years)	0	CGMH
				Female (16)					
Nasopharyngeal carcinoma	6/2015 – 6/2018	(42)	IMRT:56Gy/28 fractions	Male (13)	48	94.9% (3 years)	85.2% (3 years)	0	SPHIC
				Female (56)					
			IMCT:15-17.5GyE/5 fractions						
Locally recurrent nasopharyngeal carcinoma	5/2015 – 6/2019	(38)	CIRT: 50-69GyE (2.0-3.0 GyE/daily fraction)	Male (53)	49	83.7% (2 years)	58.0% (2 years)	Less than 20%	SPHIC
				Female (153)					
Tracheobronchial adenoid cystic carcinoma	3/2016 - 12/2019	(40)	66-72.6 GyE in 10-22 fractions	Male (10)	48	100% (2 years)	100% (2 years)	1	SPHIC
				Female (8)					
Gliomas	6/2015 – 10/2018	(50)	PBT: 60 GyE/30 fraction	Male (30)	54.5	87.8% (1 years)		0	SPHIC
				Female (20)					
			PRT: 50 GyE/25 fraction + CIRT 10 GyE/5 fraction then 12 GyE/4 fraction						
Rectal cancer	7/2015 – 4/2019	(51)	CIRT: < 66Gy (RBE) (9)	Male (19)	53	82.9% (1 years)	90.4% (1 years)	3	SPHIC
				Female (6)					
			≥ 66Gy (RBE) (16)						
						65.1% (2 years)	71.8% (2 years)		
Pancreatic cancer	5/2015 – 7/2016	(52)	62.4-68.4 GyE/32-34 fractions	Male (7)	66	80.0% (1 years)	66.7% (1 years)	1	SPHIC
				Female (3)					
						13.3% (2 years)	26.7% (2 years)		

### Prostate Cancer

Prostate cancer accounts for 26% of all incident cases and is one of the greatest number of deaths in men in 2021 (101). Prostate cancer is also very common and fast-growing in Chinese population. Recently, the Genitourinary Subcommittee of PTCOG prepared a consensus Statement on Proton therapy for prostate Cancer, which will guide the clinical practice and research direction of proton therapy for prostate cancer (8). CIRT has been used to treat clinically localized prostate cancer. According to published clinical trials and practice, CIRT showed excellent disease control and acceptable toxicity for prostate cancer (45, 102, 103).

From June 2014 to October 2019, 154 prostate cancer patients were treated in the SPHIC (104). The percentage of intermediate-, and high-risk patients was 94%. Overall survival rate and biochemical control rate is 100% and 93% in three years, respectively. Sixty-four patients with localized prostate cancer underwent CIRT to assess toxicity and quality-of-life at the SPHIC using the Expanded Prostate Cancer Index Composite-26 (EPIC-26) (45). Forty-six (71.9%) men were treated with 59.2–60.8 GyE/16 fractions, whereas 18 men (28.1%) received a prescription dose of 66 GyE/24 fractions. The incidence of acute grade 1 and 2 and late Grade 1 and 2 genitourinary toxicity was 20.3 and 10.9%, 3.1 and 1.6%, respectively. No acute or late gastrointestinal toxicity occurred. Those results are similar to the results reported in Japan (105). Because the follow-up time period is too short in SPHIC, mid-term and long-term follow-up data of efficacy and toxicity should be regularly follow-up and the results will be reported in future publications, so as to compare the toxicity and patients benefit with conventional treatment modalities.

### Head and Neck

#### Chordoma or Chondrosarcoma

Sarcomas of the base of the skull (SBS) accounts for a small proportion of head and neck malignancies. Surgery is the common treatment manner for SBS. Whereas, due to the anatomical complexity and radioresistant of SBS, the efficacy of conventional radiotherapy was weakened. Chen *et al.* (32) reported 31 patients underwent PBT of their chordoma at the WPTC. The prescribed dose was 50.4-80 GyE/28-40 fractions. With a median follow-up of 38.2 (range 6–76) months, the 3-year and 5-year OS and PFS were 90.3 and 80.4%, 87.1 and 60.5%, respectively (32).

A total of 62 patients with SBS received PBT and CIRT at the SPHIC between July 2014 and May 2019 (46). With a median follow-up of 20.4 (range 2.73–91.67) months, the 2-year OS and LRFS were 80.2% and 80.2%, respectively (39). There have two patients with Grade ≥ 3 toxicities occurred. It should be noted that these results are not the result of PBT and CIRT alone, and there is also the effect of chemotherapy.

#### Orbital Malignancies

Although orbital tumors are relatively rare, treatment is complicated because of their proximity to the critical OARs. With the rapid development of CIRT or PBT in the management of head and neck malignancies, many radiation oncologists have turned their attention to treat head and neck malignancies with CIRT or PBT (106). 22 patients with orbital malignancies received eye-sparing surgery followed by CIRT (n = 18), PBT (n = 1), or PBT plus CIRT boost (n = 3) at the SPHIC. With a median follow-up of 20.25 (range 3.8–38.8) months, the 2-year OS, PFS, and local progression-free survival (LPFS) were 100, 57.9, 92.9%, respectively. No acute severe (i.e., ≥grade 3) toxicity was observed, except for two patients with severe visual impairment (47).

#### Locoregionally Recurrent Head and Neck Malignancies

Sarcomas of the head and neck is considered a rare clinicopathological entity. It is difficult to *en bloc* surgical removal, so radiotherapy is a good choice. Salvage radiotherapy is usually limited by the adverse-effects. Although there have several publications reported the clinical outcomes in CIRT sarcoma treatment, but re-irradiation for HNS sarcoma is limited. PBT and CIRT can give full play to its advantages to extend the survival rate of patients. 141 patients with LR-HNS treated with IMCT at SPHIC (18, 107). The median dose was 60 GyE (range 50–69 GyE, 2.0–3.5 GyE/daily fraction). The 1-year overall survival rate was 95.9%, which is better than reported one-year OS rates (30–40%) (18). 7.1% of the patients reached grade ≥3 acute or late toxicities. Ten patients experienced Grade 4 hemorrhage. These results are highly analogue to patients treated at the Heidelberg Ion Therapy center (HIT) (108). It can be seen that salvage radiotherapy for HNS sarcoma may be associated with severe adverse effects and only provide palliative effects.

#### Sinonasal Malignancies

Sinonasal malignancies (SNM) accounts for 3%~5% of head and neck malignancies. The efficacy of IMRT over conventional RT for SNM remains to be controversial. The advent of PBT and CIRT brings hope for the treatment of SNM depending on their advantages in physical and radiobiological aspects (109). One-hundred-and-eleven patients received particle-beam radiation therapy (PBRT) at the SPHIC, including CIRT alone (n = 70), PBT plus CIRT boost (n = 37), and PBT alone (n = 4). The median follow-up was 20.2 months. The 2-year OS rates, PFS, and LPFS were 82, 66, and 83%, respectively. Grades 3-4 late toxicity occurred only 4 (3.6%) patients (48). A clinical experience and short-term efficacy in the management of olfactory neuroblastoma (ONB) also shown that PBRT is well tolerated and safe and effective for the treatment of ONB at the SPHIC (110).

#### Head and Neck Adenoid Cystic Carcinoma

Adenoid cystic carcinoma is a rare malignant tumor of the head and neck, commonly found in the salivary glands. Slow growth, local recurrence and distant metastasis have always been considered as typical clinical features of the disease. Eight patients with H&N ACC were treated using PBT or CIRT to study the early response and toxicities at the SPHIC (111). Seven patients received IMPT followed by IMCT boost. One of them received IMPT alone. With a 3-months follow-up, 50% patients achieved PR or CR. Except for 2 patients who experienced grade 3 mucositis, no patients experienced serious toxicities.

#### Nasopharyngeal Carcinoma

Nasopharyngeal carcinoma is one of the common malignant tumors in China, especially in the south. Local recurrence remains one of the most important modes of treatment failure in the management of patients with NPC. Radiotherapy is currently the preferred treatment for nasopharyngeal carcinoma. The benefits of CIRT in the salvage treatment of patients with locally recurrent head and neck malignancies have been reported (96). SPHIC and CGHM have achieved some exciting results in NPC radiotherapy (**Table 5**).

Between 2016 and 2018, 80 patients with NPC who received intensity-modulated proton therapy (IMPT) at the CGMH. The prescribed dose was 69.96 GyE for IMPT given in 2.12 GyE fractions. Median follow-up time was 24.1 months (18.2–34.3) for patients treated using IMPT. No patient died in the IMPT group. The 2-year overall survival (OS) rates were 100% for the IMPT. Four events (recurrence or death) were observed in the IMPT group, with a two-year progression-free survival (PFS) rate of 94.4%. Four patients (5%) treated with IMPT required NG tube placement. The mean percentage of body weight loss (BWL) during RT was 4.87% in the IMPT group (49). Propensity score matching analysis of patients treated with IMPT and Volumetric Modulated Arc Therapy (VMAT) has shown IMPT is safe and beneficial for NPC patients.

Satisfactory efficacy and acceptable toxicity of IMRT with CIRT boost has been achieved in a small group of patients (112). A large sample size of 69 NPC patients treated with mixed-beam radiotherapy using IMRT and CIRT boost at the SPHIC (42). With a median follow-up of 31.9 months, the 3-year OS, PFS, local control rates were 94.9, 85.2, 96.9%, respectively. No severe radiation-induced late toxicity was observed, except for two patients with dermatitis. Therefore, mixed photon and carbon-ion beam radiotherapy present an excellent disease control and acceptable toxicity for NPC patients.

Intensity-modulated carbon-ion therapy (IMCT) treatment of NPC has obvious advantages over IMRT in terms of serious adverse reactions (108). Kong et al. reported 206 cases of locally recurrent nasopharyngeal carcinoma treated with CIRT. The dose of salvage radiotherapy is 50-69 GyE (2.0-3.0 GyE/daily fraction) (38, 113). With a median follow-up of 22.8 months, the 2-year OS, local control, regional control, and distant control rates were 83.7%, 58.0%, 87.3%, and 94.7%, respectively. There was no case of mucosal or other radiotherapy-related adverse reactions of grade 2 or higher during follow-up. Whereas, long-term follow-up is necessary to determine the optimal dose and long-term outcome and late toxicities.

### Intracranial Tumors

ICT refers to tumors that are primary or secondary to the cranial cavity. ICT has gradually become one of the important tumor diseases that endanger the health of the Chinese population and cause death. At present, the main treatment methods for ICT include surgery, radiotherapy, chemotherapy, interventional therapy, and targeted therapy. Radiotherapy is the standard adjuvant therapy to intracranial tumor after surgery (114). Due to the complicated histological subtypes of brain tumor, the clinical outcomes will be presented in two parts.

#### Gliomas

Glioma ranks first among adult central nervous system malignant tumors. The tumor progresses rapidly, the recurrence rate after treatment is high, and the cure rate is low, making it a major medical challenge. Surgical resection is the main treatment method. Due to its biological characteristics of invasive growth, surgery is difficult to completely remove, and it is prone to recurrence after surgery, so postoperative radiotherapy is often used as adjuvant treatment. From December 2004 to October 2006, 46 cases of gliomas were treated at the WPTC (33). The total dose ranged from 45 to 72 GyE and each fraction dose from 1.8 to 5 GyE in malignant tumor. The follow-up period for glioma patients range from 1 to 20 months (averaged 9.8 months). Thirteen patients showed disappearance of the tumor (27.1%), 29 cases with PR (60.4%).

Between May 2015 and October 2018, 50 patients with histology confirmed high-grade gliomas (HGG) received either PBT or PBT with a CIRT boost at the SPHIC (50). All patients received temozolomide. Twenty-four patients received PBT at a dose of 60 GyE/30 fractions, and 26 patients received PBT plus a CIRT boost in various protocols. At a median follow-up of 14.3 months (range, 4.8-39.6 months), the 12-month and 18-month OS rates were 87.8% and 72.8%, respectively, and the 12-month and 18-month PFS rates were 74.2% and 59.8%, respectively. Twenty-nine patients experienced grade 1 treatment-related acute adverse effects, and 11 developed grade 1 (n = 6) or grade 2 (n = 5) late adverse effect of radiation-induced brain necrosis. No grade 3, 4, or 5 toxicities were observed. To assess the effect of CTRT boost after PBT, a randomized trial is recruiting patients (37).

#### Meningioma

Meningioma is one of the most common intracranial tumors, accounting for about 33% of central nervous system tumors (115). PBT has a better dose distribution than conventional photon therapy, so it can improve tumor control and better protect normal brain tissue (116). Twenty-six patients with meningioma treated with PBRT from May 2015 to October 2018 at the SPHIC. The median dose was 54 GyE (range 50.4-60 GyE, 1.8-2 GyE/daily fraction). With a median follow-up of 22.2 (range 1.6-36.4) months, the 2-year overall survival and progression-free survival rates were both 100%. Grade I skin erythema and alopecia were observed in 22 patients and Grade I mucositis was observed in 2 patients. No acute of late toxicities of Grade 2 or above was observed (117). The safety and favorable toxicity profile of CIRT for meningioma also were confirmed at many institutes (118).

### Thoracic Cancers

Thoracic tumors are the most morbidity sites in China, and radiotherapy is one of the main treatments for chest tumors, including Lung cancer, breast cancer, esophageal cancer, etc. While thoracic radiotherapy kills tumors, it often causes radiation-induced lung damage, which limits the further increase in the dose of radiotherapy to eliminate tumors and also seriously affects the quality of life of patients (119).

#### Lung Cancer

According to global cancer statistics 2021, lung cancer is the leading cause of death among males (101). The highest incidence rates among men are observed in China (rates are above 40 per 100,000). Currently, as for those patients who are not suitable for surgical resection, radiotherapy is still an important and potentially treatment for local and recurrent non-small cell lung cancer (NSCLC) (120).

From August 2014 to December 2015, 10 patients with stage I non-small cell lung cancer (NSCLC) who were inoperable or refused surgery were treated by proton alone or proton combination with carbon-ion RT to evaluate the safety and efficacy of PBT and CIRT for stage I NSCLC with pencil beam scanning technique at the SPHIC (121). The prescribed dose was 50-72 GyE with 10-24 fractions. With the median follow-up of 18.1 (11.9-28.1) months, local control was found in all patients with 6 complete response (CR), 3 partial response (PR), and 1 stable disease (SD). No Grade ≥ 3 toxicity occurred in all patients. Two patients occurred grade 2 toxicities with acute skin reaction and leucopenia.

#### Tracheal Adenoid Cystic Carcinoma

TACC is a kind of tumor with low incidence and not easy to be found. Although it has been reported that radiotherapy can be used for the treatment of TACC, its specific role is unknown (122). Eighteen patients with TACC were treated using CIRT at the SPHIC. The patient received 66–72.6 GyE/22–23 fractions. During a median follow-up period of 20.7 (range 5.8–44.1) months, 3 patients developed lung metastasis, and one of them experienced local recurrence. The lung metastasis rate is consistent with previous reports (123).The rates of 2-year OS, LC, and PFS were 100, 100, and 61.4%, respectively. Only one patient experienced grade 4 tracheal stenosis, no other grade ≥ 3 adverse effects were observed (40).

### Hepatocellular Carcinomas

Hepatocellular carcinoma (HCC) is a type of malignant tumor with high morbidity and mortality. In China, the total number of HCC patients accounts for more than half of the world’s cases every year (124). Most patients are associated with chronic viral hepatitis B infection and cirrhosis (125). Although surgery or radiofrequency ablation can cure hepatocellular carcinoma, few patients are operable due to advanced disease or medical comorbidities. Due to the high radiosensitivity of normal liver tissue and respiration motion of patient, RT was very limited for liver cancers. To reduce the unwanted effect in HCC RT, some methods have been developed, especially respiratory control and image guidance technology, which can achieve encouraging responses and only minimal toxicities (126–128).

Between 2005 and 2007, a total of 32 patients with primary hepatocellular carcinoma have been treated with PBT at the WPTC in ZIBO, China (34). PBT was delivered to a total dose of 60 -70 GyE in 7 fractions. In this study, 9 cases achieved clinical CR, 37 cases achieved PR and 3 cases suffered SD. The objective therapeutic effect is approximately 90.6%. According to Karnofsky Performance Status (KPS) score, 10 cases improved the quality of life (31.25%). The improvement rate of liver function reached 53.13%. Adverse reactions were classified according to Radiation Therapy Oncology Group (RTOG) criteria for acute radiation injury, including 0 Grade (16 cases), 1 Grade (12 cases), 2 Grade (3 cases), 3 Grade (1 cases), 4 Grade (0 cases). Proton therapy can not only improve the quality of life of patients, which may indicate reduced toxicity with PBT.

From January 2007 to January 2018, a total of 55 patients treated with PBT were enrolled for primary hepatocellular carcinoma at CGMH. According to the tumor location, 72.6 GyE in 22 fractions or 66 GyE in 10 fractions were prescribed. Through a propensity-matched analysis, compared with photon radiotherapy, significant survival benefit in the proton group and lower risk of RILD (11.8% vs. 36%, *p* =0.004) was achieved (53). Between November 2015 and December 2017, 30 patients with unresectable cholangiocarcinoma (CC) had undergone PBT. The median radiation dose was 72.6 GyE. The median OS and PFS were 19.3 and 10.4 months, respectively. Seven percentage of patients suffered acute skin reactions. Three patients had grade ≥ III toxicities and two patients had radiation-induced liver disease (129).

### Skin Malignant Neoplasm

The incidence of skin cancers has continuously risen. Although surgery is the preferred strategy, radiation therapy is recommended for tumors that are difficult to remove, for example, primary or lymph node-invaded melanoma. Most of CIRT are focused on deep-seated tumors, but treatment of skin carcinomas is limited. Between November 2006 and March 2009, forty-five patients with superficial carcinoma were treated with CIRT at the IMP (55). The range of total dose was 60 to70 GyE for squamous cell carcinoma (SCC) and basal cell carcinoma (BCC), 61–75 GyE for malignant melanoma (MM), 60 GyE for Bowen disease and 42.5 GyE for Paget disease, administered in 6-11 fractions within 6-11 days. The single dose was 7-10 GyE. The median follow-up period was 24 months (range,12-36 months) and the follow-up rate was 100%. The 3-years actuarial local control rates were 65.5% for SCC, 80.2% for BCC, 42.9% for MM, 90% for Bowen and Paget diseases, respectively. The actuarial 1- and 3-year overall survival rates reached 86%. No severe side-effects greater than grade 3 have been observed.

### Keloids

Keloid is a benign tumor of the skin, but it has the characteristics of malignant tumor infiltrating into the surrounding normal tissues (130). Keloid is accompanied by chronic inflammation, which is prone to infection and ulceration. At present, the main treatment for keloids is surgical resection, but there is a high recurrence rate after surgical resection alone, so it is often necessary to combine other adjuvant treatments after surgical resection (131). Surgical resection combined with radiotherapy is a more conventional treatment for keloids. At present, the principle of radiotherapy for keloids is still unclear. Recently, 16 patients with keloids received postoperative CIRT at the IMP. The prescribed dose was 16GyE/8 fractions (56). With a medium follow-up of 29.7 months (range 24.3­35.3 months), 95% success rates were achieved. No grade 3 or higher toxicity and complication occurred. As far as we know, this should be the first report of CIRT for keloids. This result indicates that surgery combined with CIRT can be used in the treatment of keloids in the future (56).

### Rectal Cancer

Rectal cancer is one of the common gastrointestinal malignancies. Due to the lack of specific symptoms of rectal cancer, many patients have undergone local infiltration or distant metastasis. Total mesorectal excision (TME) after preoperative neoadjuvant chemoradiotherapy has become the main treatment manner for patients with locally advanced rectal cancer. Many clinical studies have demonstrated that preoperative radiotherapy can improve local control rate and reduce the risk of recurrence. Twenty-five patients with unresectable locally recurrent rectal cancer (LRRC) were treated by CIRT from July 2015 to April 2019 at SPHIC (51). The LC rates at 1 and 2 years were 90.4 and 71.8%. The OS rates at 1 and 2 years were 82.9 and 65.1%. No Grade 4 or higher toxicity was observed. In contrast to National Institutional of Radiological Sciences Hospital (NIRS) (132), there was a good correlation between prescribed dose and local control rate.

### Pancreatic Cancer

Pancreatic cancer has a high mortality rate, and 80% to 85% of patients present with locally advanced or distant metastases. Radiotherapy plays an important role in the treatment of pancreatic cancer and has achieved encouraging therapeutic effects. A phase I dose escalation study of PBT and CIRT has been carried out for the patients with locally advanced pancreatic cancer (LAPC) at SPHIC (52). The proton dose of 50.4 GyE and carbon ion as a boost dose varying from 62.4 GyE/32 fractions to 68.4 GyE/34 fractions were delivered. With a median follow-up of 17.4 months, no patient developed dose limiting toxicity, and 40% of patients suffered from acute gastrointestinal (GI) and hepatic toxicity. The OS rates at 1 and 2 years were 80.0% and 13.3%. It is worth noting that only ten patients were enrolled in this study. Therefore, more patients and long-term follow-up are needed to determine the outcome of treatment. According to NIRS reports, the OS rates at 1 and 2 years were 73% and 35% (133). The difference may be due to different prescribed dose and combination modality of radiotherapy.

## Future Challenges

Over the past decade there has been considerable interest and progress in the use of protons and carbon in an attempt to improve the effectiveness of radiotherapy in China. Considering the actual situation in China, it is necessary to strengthen the talent training of medical physicists and develop advanced radiotherapy technology and supporting equipment such as rotating gantry. These centers should coordinate and integrate all social resources to promote market application as soon as possible (134). Small sample size and high heterogeneity are the common features for particle radiotherapy in China. Owing to the length of time needed to accumulate epidemiologic data, a concerted multicenter, international effort should be established for long-term follow-up of charged particle RT patients in China. Although China has been in the forefront of proton and heavy ion therapy in the world, and has achieved some encouraging results in the treatment of cancer, the five proton or heavy ion treatment centers in China are only expert in a few types of cancer. Other types of cancer treatment need long-term exploration and optimization to achieve the best therapeutic effect. The incidence of cancer in China is very different from that in Europe, America and Japan (1), for instance, the incidence of breast cancer ranks first in the world, but ranks fourth in China after lung cancer, colorectal cancer and stomach cancer. Therefore, proton and heavy ion medical facilities in China should pay more attention to the more common types of cancer in China and actively explore new treatment technologies. The early data suggest that carbon ion radiotherapy provides favorable local tumor control and overall survival with acceptable rates of late complications. In addition, some challenges are the same as other particle therapy centers around the world, for instance, long-term follow-up of patients after treatment is necessary in order to compare with other conventional treatments and to highlight their advantages.

## Discussion and Conclusion

China has the largest population and ranks first in the number of new cancer cases and deaths in the world (135), but the number of PBT and CIRT centers in operation in China is very small. Early PBT and CIRT results are satisfactory in China. However, we need to interpret the data with caution. Domestically, there is still no comparative analysis of the therapeutic effects of the three radiotherapy methods among IMRT, PBT and CIRT. Therefore, there is still a long road for researchers and physicians to explore the advantages of PBT and CIRT.

Tumor cells treated with carbon ions sensitized to several chemotherapeutic drugs (carboplatin, paclitaxel, and etoposide) (136). Until now, several clinical trials are available on the combination of chemotherapy and PBT or CIRT (137). Only one clinical trial to evaluate the efficacy of CIRT with concurrent cisplatin for LR-NPC at the SPHIC was registered (NCT02801487). In addition, radiotherapy fractionation for different tumor sites and sequential treatment of chemotherapy and radiotherapy is also important for the treatment outcome (138, 139). The same is true in China, where more clinical trials are needed to determine the optimal fractional dose and treatment sequence. RT is a particularly promising candidate for combination with immune checkpoint blocker (ICB) (140). A search for “radiotherapy plus immunotherapy” on ClinicalTrials.gov yielded 217 results (141). Indeed, radiotherapy not only has a direct killing effect on tumor cells, but also reprograms the tumor microenvironment to exert an effective anti-tumor immune response (142). The new treatment strategy of radiotherapy combined with immunotherapy has achieved remarkable curative effect on a variety of tumor models. Preliminary clinical trials have shown that this new therapy has achieved good results for patients with metastatic solid tumors, especially breast cancer, prostate cancer and NSCLC (143). Thus, combining radiotherapy and immunotherapy is a crucial strategy to improve patient survival (144). More detailed discussion of the mechanisms of CIRT and combination immunotherapy can be found in several review articles (145, 146). We have reason to believe that clinical trials combining radiotherapy and immunotherapy will be carried out in China in the next few years.

Although particle radiotherapy is a promising treatment method, the cost-efficiency ratio is an urgent problem to be solved (134, 147). Although heavier ions have the potential to significantly improve clinical results for radio-resistant indications and especially hypoxic tumors, carbon ion facilities cost about 2–3 times more than proton facilities. Building CIRT centers is also complicated. Design of these facilities should take into consideration the treatment delivery method, number of rooms needed, choice of gantry (or not), and the expected number of fractions or patients to be treated annually. To maximize the advantage of PBT and CIRT and minimum the costs, physicists and physicians should strengthen collaboration. More attention should be paid for physical technical, beam-delivery systems and treatment technology. Many improvements are already in progress in China. More recently, a variable cycle-based respiratory guidance method for moving target treatment was developed at the IMP, which will improve the CIRT treatment efficiency and precision for organs that move with breathing (127). In addition, a fast 3D rescanning method allows for 3D scanning that is about 100 times faster than conventional systems (148). Image-guided radiation therapy (IGRT) directs irradiation utilizing the imaging coordinates of the actual treatment plan, has further improved the accuracy and safety (149). Therefore, these two advanced technologies are the focus and difficulty of future research and development in China. In order to more conveniently carry out all-round RT for patients, a superconducting rotating gantry is also being developed at the IMP to reduce the footprint occupied by the equipment as much as possible.

There is an unmet need for comparative treatment planning study among different radiotherapy manners for patients to assess the potential benefits and limitations of different treatment modalities (11). In addition, there have been a number of preclinical studies based on cells and animals showing that ultra-high dose rate (FLASH) irradiation reduces damage to normal tissues while preserving the ability to treat tumors (150). As traditional therapeutic devices cannot meet the technical and safety requirements of FLASH radiotherapy, there are no publicly reported domestic studies on FLASH radiotherapy. However, with the successful application of FLASH radiotherapy in the first tumor patient (151), we believe that China will also pay attention to the development of new radiotherapy technologies in the future.

With the development of whole genome sequencing technology, the era of personalized therapy has arrived, which can extend to radiotherapy (152). There is increasing evidence that genetic mutations are closely related to radiosensitivity of tumors (153). PBT and CIRT centers are especially in short supply in China. In order to maximize the treatment efficiency of patients and save resources, we should consider detecting genetic mutations in patients in the future (154).

The Chinese health care system and specifically, radiation oncology, has clearly improved during the past 30 years in equipment and its use, although the shortage of facilities and workforce remain to be improved. In addition to the rapid increase in the number and quality of the facilities in China, the training of radiation oncologists and medical physicists (radiation oncology residency programs) also improved (134). Unresectable sarcoma became the only tumor type where CIRT is covered under the national healthcare insurance in Japan since 2016 (155). In China, the cost of particle therapy is borne by the patient themselves. The Chinese healthcare reimbursement is expected in the near future with cancer sites expected to be covered by the national insurance (134). At present, the SPHIC adopts a method of cooperation with commercial insurance.

There are too few facilities to conduct proton vs carbon prospective and randomized clinical trials required to compare the two modalities (156). Meanwhile, multi-institutional analysis studies of CIRT for different cancer sites is rare due to the small number of CIRT institutions in the world (157). Multi-institutional analysis can demonstrate standard and average treatment outcomes that can be utilized as reference in China. At present, there are several multi-institutional retrospective studies to evaluate the efficacy and safety of proton or carbon-ion radiotherapy (158–163). A randomized controlled trial comparing IMRT, PBT and CIRT would require a very large patient cohort to show significant differences in local control and/or toxicity. Hence, the multicenter international Radiation Oncology Collaborative Comparison (ROCOCO) was initiated in 2007 conducting several comparatives *in silico* trials in multiple primary tumor sites (164–166). In addition, physical therapist for tumor radiotherapy should adopt the best modality to specific tumor individuals according to multi-institutional analysis.

In short, although particle therapy in China is in the stage of rapid development, it is believed that particle therapy in China will be bright with the cultivation of domestic talents and the maturity of technology.

## Author Contributions

YL selected the references, wrote the text, and approved the final version of this manuscript. JY, XL, MT, YG, SW, and JX contributed to discussing the content, review and editing of manuscript before submission. All authors contributed to the article and approved the submitted version.

## Funding

This work was supported by grants from Guangdong Innovative and Entrepreneurial Research Team Program (No. 2016ZT06G373).

## Conflict of Interest

The authors declare that the research was conducted in the absence of any commercial or financial relationships that could be construed as a potential conflict of interest.

## Publisher’s Note

All claims expressed in this article are solely those of the authors and do not necessarily represent those of their affiliated organizations, or those of the publisher, the editors and the reviewers. Any product that may be evaluated in this article, or claim that may be made by its manufacturer, is not guaranteed or endorsed by the publisher.

## Abbreviations

RBE, relative biological effectiveness; WPTC, Wanjie Proton Therapy Center; SPHIC, Shanghai Proton Heavy Ion Center; HIMM, Heavy Ion Cancer Treatment Center; CGMH, Chang Gung Memorial Hospital; RT, radiotherapy; PTCOG, Particle Therapy Co-Operative Group; PBT, proton beam therapy; CIRT, carbon ion radiotherapy; LET, linear energy transfer; LR-NPC, locally recurrent nasopharyngeal carcinoma; OER, Oxygen Enhancement Ratio; IMP, Institute of Modern Physics; HCC, Hepatocellular carcinoma; OS, overall survival; PFS, progression-free survival.
