# Supplementary material for: Flourish of Proton and Carbon Ion Radiotherapy in China
Source: Front Oncol. 2022 Feb 14;12:819905. doi: 10.3389/fonc.2022.819905 (PMC8882681; doi:10.3389/fonc.2022.819905)
Supplement: Supplementary file 1 [file Table_1.docx]

Supplementary Material

# Supplementary Data

**Table S1** **Particle therapy facilities under construction in China (update Jan. 2022)**

| **Centers** | **Location** | **Sources of equipment** | **Type of Particle** | **Beam directions** | **No. of treatment rooms** | **Start of construction** | **Medical device approval** |
| --- | --- | --- | --- | --- | --- | --- | --- |
| HITFil at IMP, | Lanzhou, Gansu | Self-developed | Carbon | 4 horiz, vertical, oblique, fixed beams | 4 | 2012 | Yes |
| Zhuozhou Proton Therapy Center | Baoding, Hebei | IBA | Proton | 4 gantries,   1 horiz. fixed beam | 5 | 2015 | Yes |
| Guangdong Hengjian Medical Technologies | Guangzhou | IBA | Proton | 3 gantries | 3 | 2014 | No |
| Qingdao Zhong Jia Lian He Healthcare | Shandong | IBA | Proton | 4 gantries, 1 fixed beam | 5 | 2016 | No |
| Beijing Proton Center | Beijing | IBA | Proton | 3 gantries, 1 horiz. fixed beam | 4 | 2017 | Yes |
| Hefei ion medical center | Hefei, Anhui | Varian Probeam® | Proton | 3 gantries, 1 horiz. fixed beam | 4 | 2015 | No |
| Wuhan Union Hospital PTC | Wuhan, Hubei | Varian Probeam® | Proton | 2 gantries, 1 horiz. fixed beam | 3 | 2020 | Yes |
| Proton Clinical Research Center of Shandong Cancer Hospital | Jinan | Varian Probeam® | Proton | 3 gantries, 1 horiz. fixed beam | 4 | 2018 | Yes |
| Guangzhou Concord Cancer Center, GCCC | Guangzhou | Varian Probeam® | Proton | 4 gantries | 4 | 2017 | No |
| Taipei Veterans General Hospital's Heavy Ion Therapy Center | Taipei | Hitachi | Carbon | 2 vertical and 2 horizontal  fixed beams | 2 | 2019 | Yes |
| National Taiwan University | Taipei | Varian Probeam® | Proton | 2 gantries, 1experimental room | 3 | 2008 | Yes |

**Table S2 Particle therapy facilities in planning stage in China (update Jan. 2022)**

| **Centers** | **Location** | **Sources of equipment** | **Type of Particle** | **Beam directions** | **No. of treatment rooms** | **Medical device approval** |
| --- | --- | --- | --- | --- | --- | --- |
| Hong Kong Sanatorium and Hospital PTC | Hong Kong | Hitachi | Proton | 2 gantries | 2 | Yes |
| Tianjin Taishan Cancer Hospital, Sino-US proton treatment & research center | Tianjin | Provision | Proton | 2 gantries  1 fixed beam | 3 | No |
| Boao Evergrande International Hospital | Hainan | ProTom International | Proton | 2 gantries, 1 fixed beam | 3 | No |
| Jinshazhou Hospital | Guangdong | Mevion | Proton | 1 gantry | 1 | No |
| Himed Cancer Hospital | Xuzhou, Jiangsu | Hitachi | Proton, Carbon | 1 gantry (Proton), 3 fixed beams (Carbon) | 4 | No |
| Shenzhen Tumor Hospital | Shenzhen, Guangdong | IBA ProteusPlus | Proton | 4 gantries,1 fixed beam | 5 | Yes |
| Sichuan Cancer Hospital | Chengdu, Sichuan | IBA ProteusPlus | Proton | 3 gantries, 1 fixed beam | 4 | Yes |
| Jiangxi Cancer Hospital | Jiangxi | Mevion | Proton | 1 gantry | 1 | No |
| StarKids Children’s Hospital | Shanghai | Mevion | Proton | 1 gantry | 1 | No |
| Chung Shan Medical University Hospital | Taichung | Mevion | Proton | 1 gantry | 1 | Yes |
